# Supplementary material for: Post-translational coordination of chlorophyll biosynthesis and breakdown by BCMs maintains chlorophyll homeostasis during leaf development
Source: Nat Commun. 2020 Mar 20;11:1254. doi: 10.1038/s41467-020-14992-9 (PMC7083845; doi:10.1038/s41467-020-14992-9)
Supplement: Supplementary file 1 — Supplementary Information [file 41467_2020_14992_MOESM1_ESM.pdf]

Supplementary Information for

Post-translational coordination of chlorophyll biosynthesis and breakdown by BCMs maintains chlorophyll homeostasis during leaf development

Peng Wang[1*], Andreas S. Richter[1,3], Julius R. W. Kleeberg[2], Stefan Geimer[2], Bernhard Grimm[1*]

[1] Humboldt-Universität zu Berlin, Institute of Biology/Plant Physiology, Philippstraße 13, 10115 Berlin, Germany

[2] Universität Bayreuth, Zellbiologie/Elektronenmikroskopie, 95440 Bayreuth, Germany

[3] Humboldt-Universität zu Berlin, Institute of Biology/Physiology of Plant Cell Organelles, Philippstraße 13, 10115 Berlin, Germany

[*] Corresponding author. E-mail: wangp2014@gmail.com. (P.W.); bernhard.grimm@rz.hu-berlin.de (B.G.)

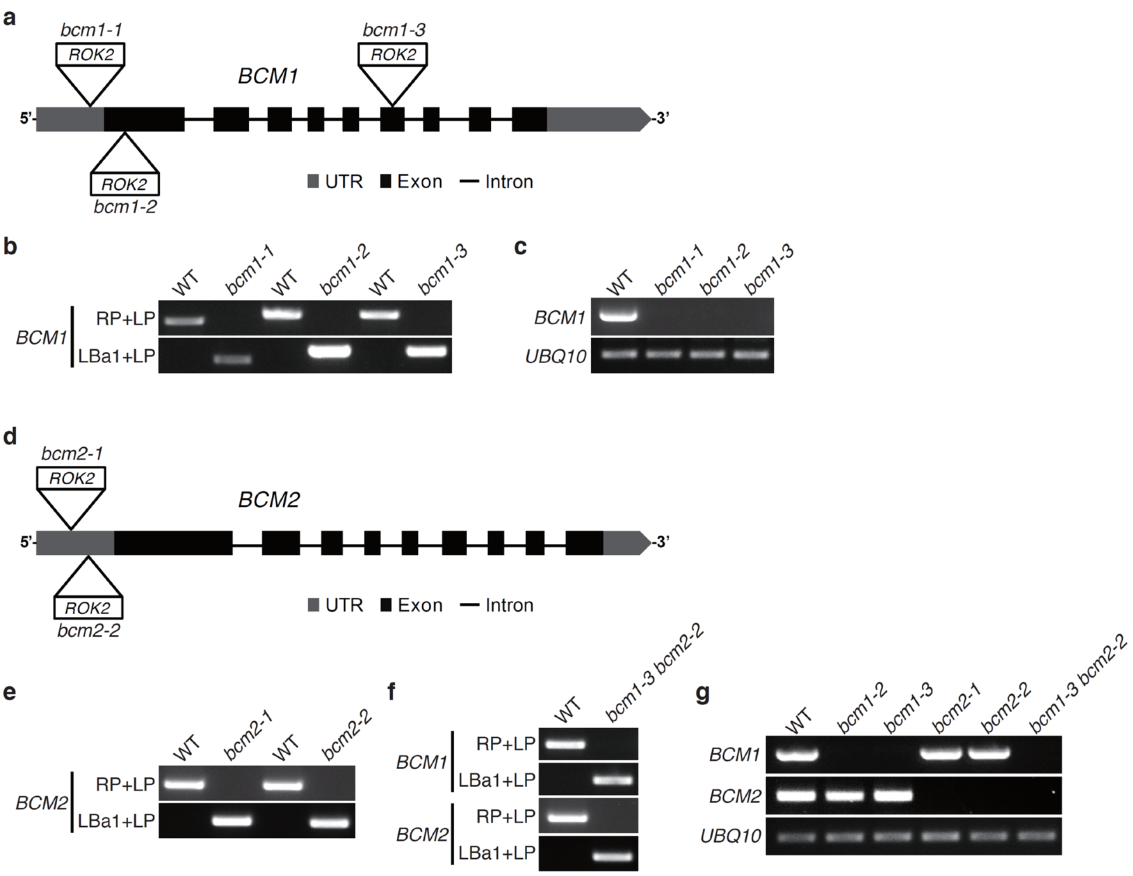

Supplementary Fig. 1. Characterization of the *BCM1* and *BCM2* T-DNA mutant lines. **a** Schematic gene structure of the *BCM1* locus (*At*2g35260) in *Arabidopsis* and positions of the T-DNA insertions in the mutant lines. The 5'-/3'-untranslated regions (UTR) and protein-coding exons are represented by light gray and dark gray boxes, respectively; introns are indicated by black lines between the boxes. T-DNAs are not drawn to scale. **b** Genotyping analyses of wild-type (WT) and homozygous *bcm1* mutant seedlings. **c** Expression of *BCM1* in WT and *bcm1* seedlings. **d** Schematic gene structure of the *BCM2* locus (*At*4g17840) in *Arabidopsis* and positions of the T-DNA insertions in the mutant lines. **e** Genotyping analyses of WT and homozygous *bcm2* seedlings. **f** Genotyping analyses of WT and homozygous *bcm1 bcm2* seedlings. **g** Expression of *BCM1* and *BCM2* in WT and *bcm1 bcm2* seedlings. In (**b**), (**e**) and (**f**), genomic PCR was performed with primers specific for the coding sequences of *BCM1* and *BCM2* (RP, right border primer; LP, left border primer) and with T-DNA-specific primer (LBa1, T-DNA left border primer 1). In (**c**) and (**g**), semi-quantitative PCR (35 cycles) was performed with primers specific for *BCM1*, *BCM2* and *UBIQUITIN 10* (*UBQ10*, as a reference gene).

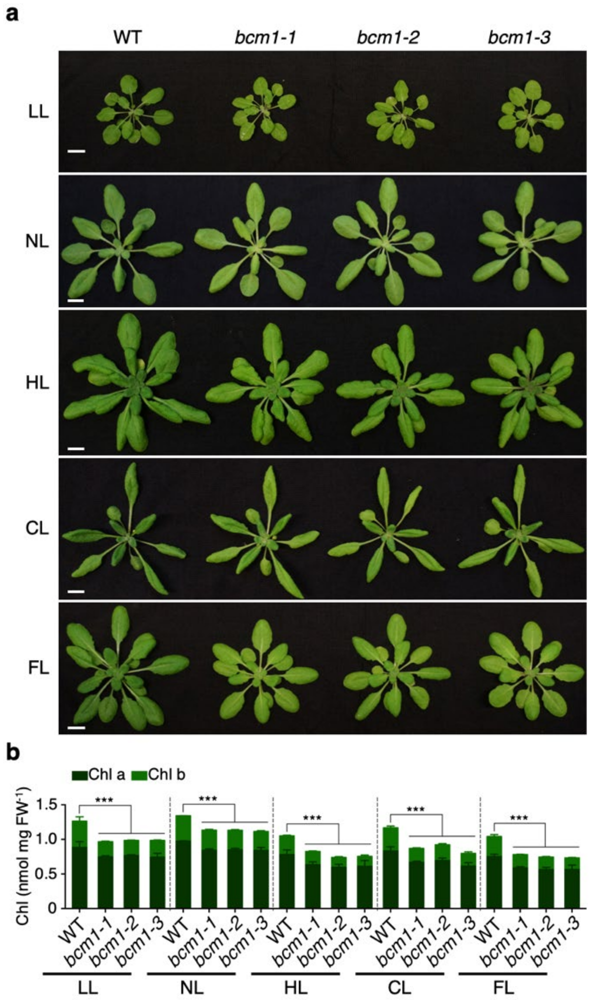

Supplementary Fig. 2. The *bcm1* mutants exhibit pale-green leaf phenotype under various light conditions.

a Representative images of 4-week-old WT and three *bcm1* seedlings grown under various light conditions, including short-day (10 h light/14 h dark) low light (LL, 30 μmol photons m^{-2} s^{-1}), short-day normal light (NL, 120 μmol photons m^{-2} s^{-1}), short-day high-light (HL, 300 μmol photons m^{-2} s^{-1}), constant light (CL, 80 μmol photons m^{-2} s^{-1}), and short-day fluctuating light (FL, 10 min low light [30 μmol photons m^{-2} s^{-1}] and 5 min high-light [300 μmol photons m^{-2} s^{-1}]). Scale bars, 1 cm. **b** Chl levels in the seedlings shown in (**a**). FW, fresh weight. Error bars represent SD of four biological replicates. Asterisks denote statistically significant differences as determined by a two-tailed Student's *t* tests with a significance of $P < 0.001$.

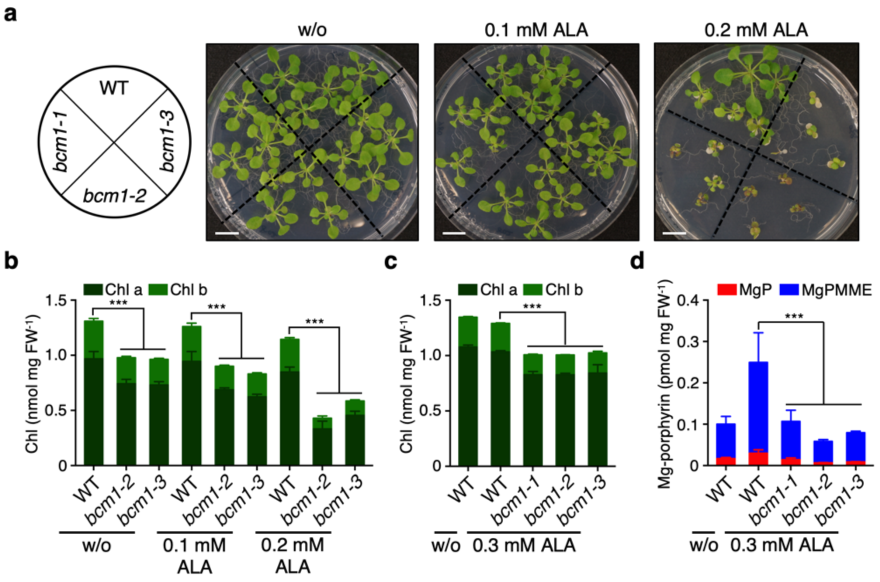

Supplementary Fig. 3. ALA feeding cannot restore pale-green leaf phenotype in *bcm1* seedlings.

a Representative images of 3-week-old WT and *bcm1* mutant seedlings grown under short-day low light (70 μmol photons m^{-2} s^{-1}) conditions on MS plates supplemented without (w/o) or with ALA. Scale bars, 1 cm. **b** Levels of Chl in the seedlings shown in (**a**). Error bars represent SD of two biological replicates. **c** and **d** Levels of Chl (**c**) and Mg-porphyrin (**d**) in the control and ALA-treated leaves. Detached leaves from 3-week-old WT and *bcm1* seedlings were incubated in PBS buffer supplemented without (w/o) or with 0.3 mM ALA for 8 hours under low light (70 μmol photons m^{-2} s^{-1}) conditions. Error bars represent SD of three biological replicates. Asterisks denote statistically significant differences as determined by a two-tailed Student's t tests with a significance of $P < 0.001$.

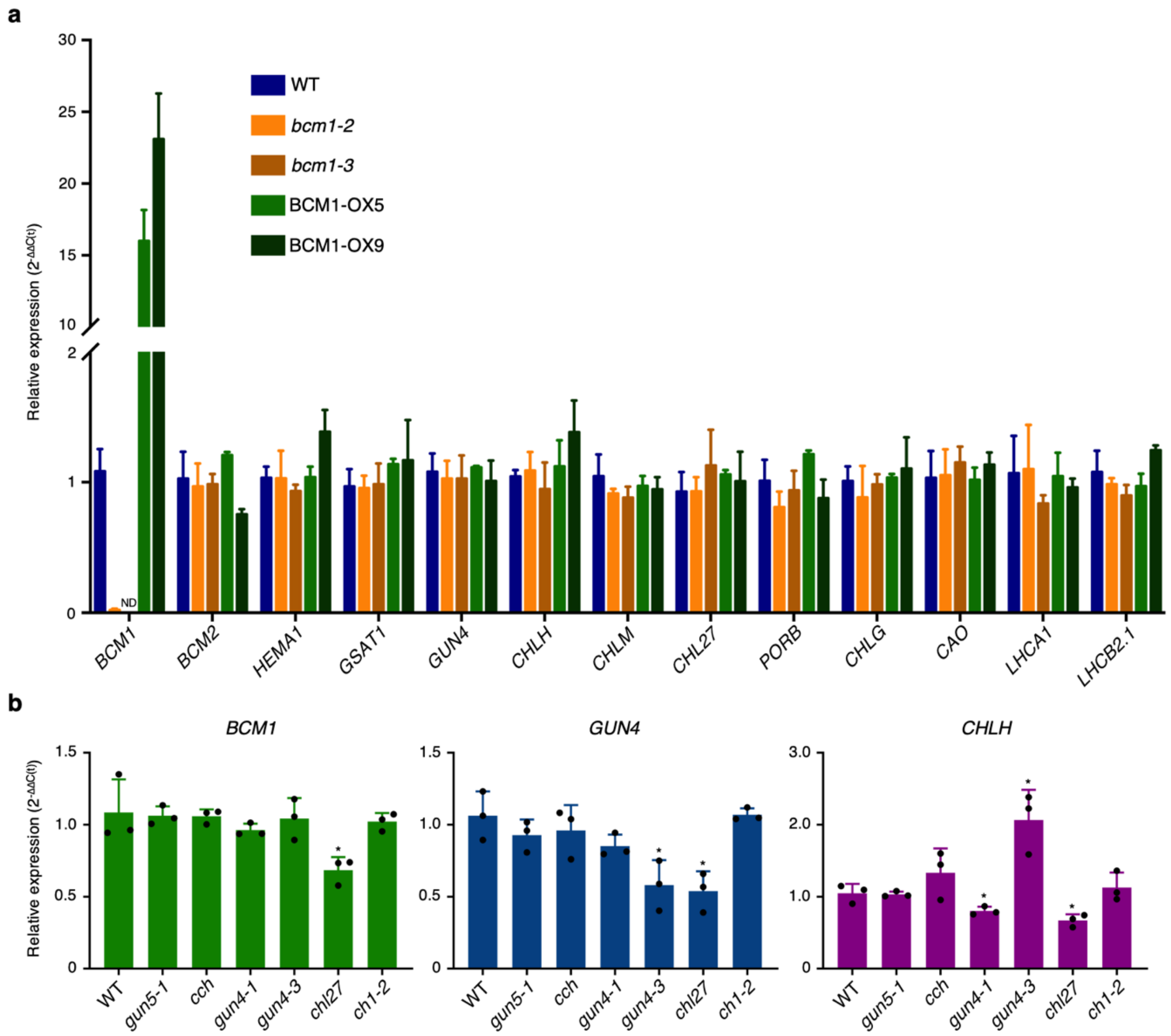

Supplementary Fig. 4. mRNA expression analysis.

a qRT-PCR analyses of the transcript levels of *BCM1*, *BCM2*, *CBG*s, *LHCA1* and *LHCB2.1* in 21-day-old WT, *bcm1*, and BCM1-OX seedlings grown under short-day normal light (120 μmol photons m^{-2} s^{-1}) conditions. Error bars represent SD of three biological replicates. **b** qRT-PCR analyses of the transcript levels of *BCM1*, *GUN4*, and *CHLH* in 28-day-old WT and mutants with deficiency of chlorophyll biosynthesis enzymes CHLH (*gun5-1* and *cch*), GUN4 (*gun4-1* and *gun4-3*), CHL27 (*chl27*) and CAO (*ch1-2*), grown under short-day low light (70 μmol photons m^{-2} s^{-1}) conditions. Error bars represent SD of three biological replicates. In (**a**) and (**b**), the relative gene expression levels of related genes were presented relative to that in WT seedlings. Asterisks denote statistically significant differences as determined by a two-tailed Student's *t* tests. **P* < 0.05.

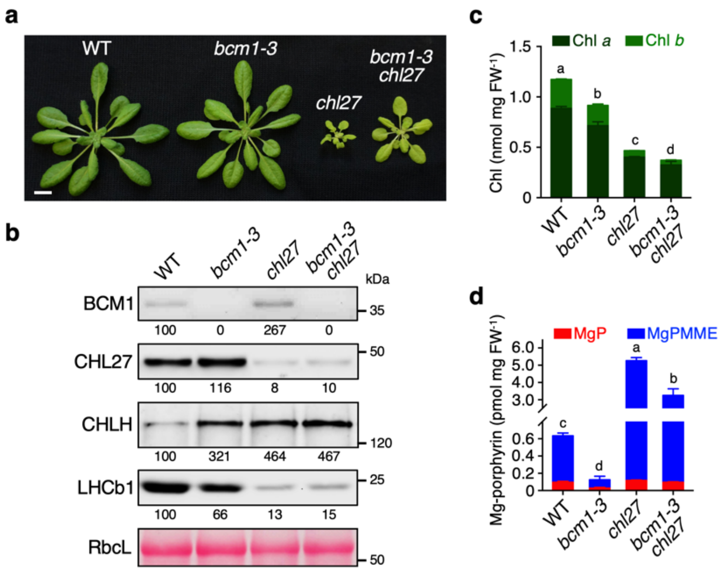

Supplementary Fig. 5. Characterization of *bcm1-3 chl27* double mutant.

a Representative images of 4-week-old WT, *bcm1-3*, *chl27* and *bcm1-3 chl27* seedlings grown under short-day low light (70 μmol photons m^{-2} s^{-1}) conditions. Scale bar, 1 cm. **b** Immunoblot analyses of indicated proteins in the seedlings shown in (**a**) using the indicated antibodies. Ponceau S-stained membrane strips bearing RbcL was used as a loading control. **c** and **d** Levels of Chl (**c**) and Mg-porphyrin (**d**) in the seedlings shown in (**a**). Error bars represent SD of three biological replicates. Letters above histograms indicate significant differences as determined by Tukey's HSD method ($P < 0.05$).

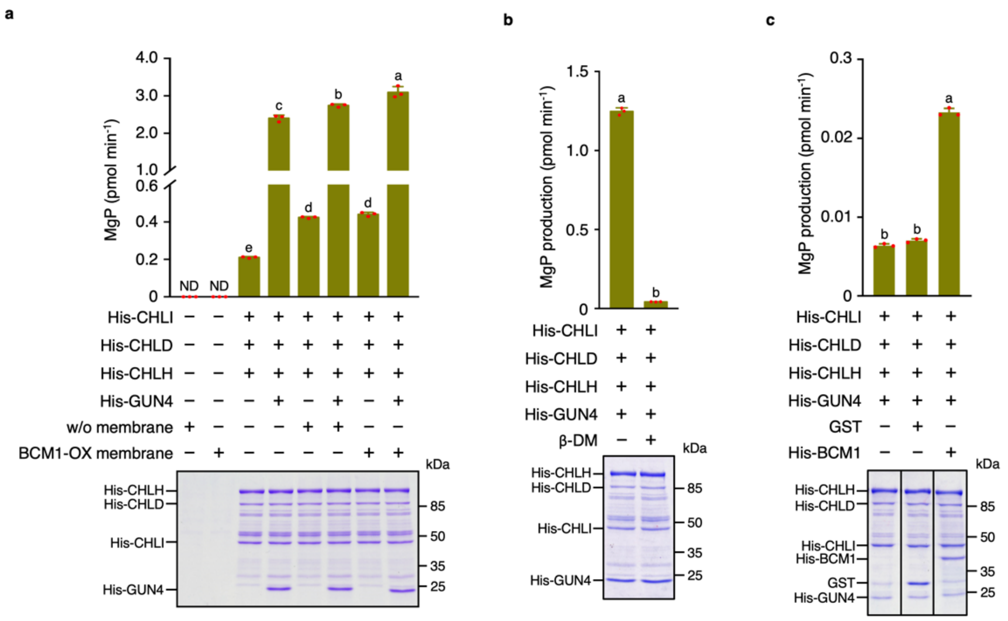

Supplementary Fig. 6. In vitro MgCh assay.

a In vitro MgCh assay with the isolated yeast membranes with (BCM1-OX membrane) or without (w/o membrane) His-BCM1. ND, not detected. **b** Addition of 0.15 mM β-DM greatly abolishes MgCh activity. In vitro MgCh assay was conducted without (w/o) or with 0.15 mM β-DM. **c** Recombinant His-BCM1 stimulates MgCh activity. GST was used as a negative control for BCM1. Production of MgP in the assay was measured by HPLC and quantified relative to incubation time. Recombinant proteins used in the assay were stained with Coomassie Brilliant Blue. Error bars represent SD of three biological replicates. Letters above histograms indicate significant differences as determined by Tukey's HSD method ($P < 0.05$).

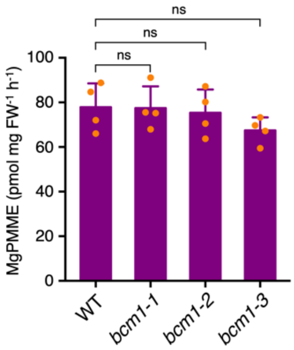

Supplementary Fig. 7. Deficiency of BCM1 does not interfere with CHLM activity in vivo.

Production of MgPMME in the assay was measured by HPLC and quantified relative to FW and incubation time (h, hour). Error bars represent SD of four biological replicates. ns, no significant difference between WT and *bcm1* seedlings.

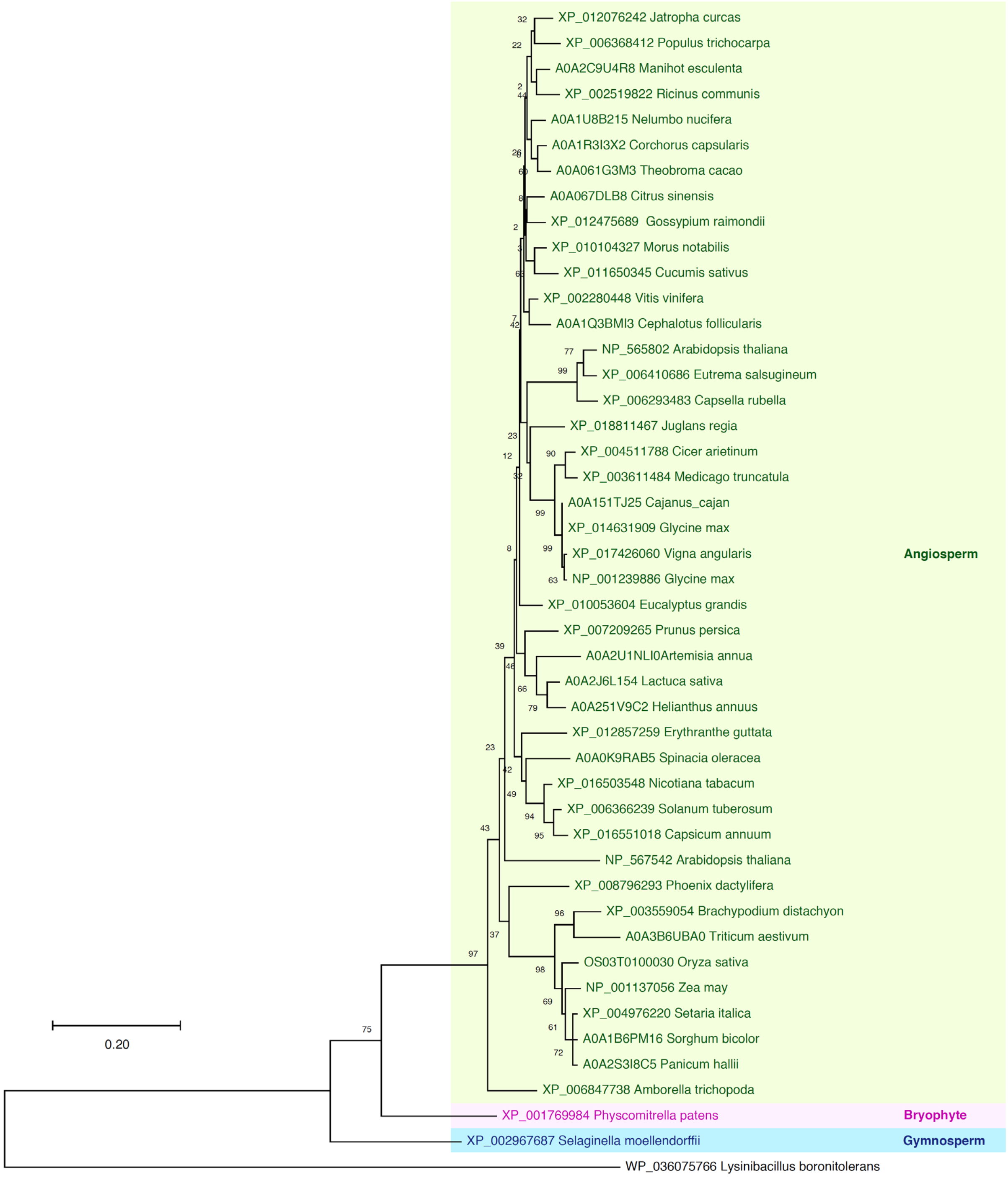

Supplementary Fig. 8. Phylogenetic analysis of BCM1 orthologs in land plants. Full-length homologous amino acid sequences of *Arabidopsis* BCM1 proteins in angiosperm, gymnosperm, and bryophyte were selected to generate a bootstrap neighbor-joining phylogenetic tree. It is rooted by *Lysinibacillus boronitolerans*. The phylogenetic tree was constructed using the MEGA X program (www.megasoftware.net).

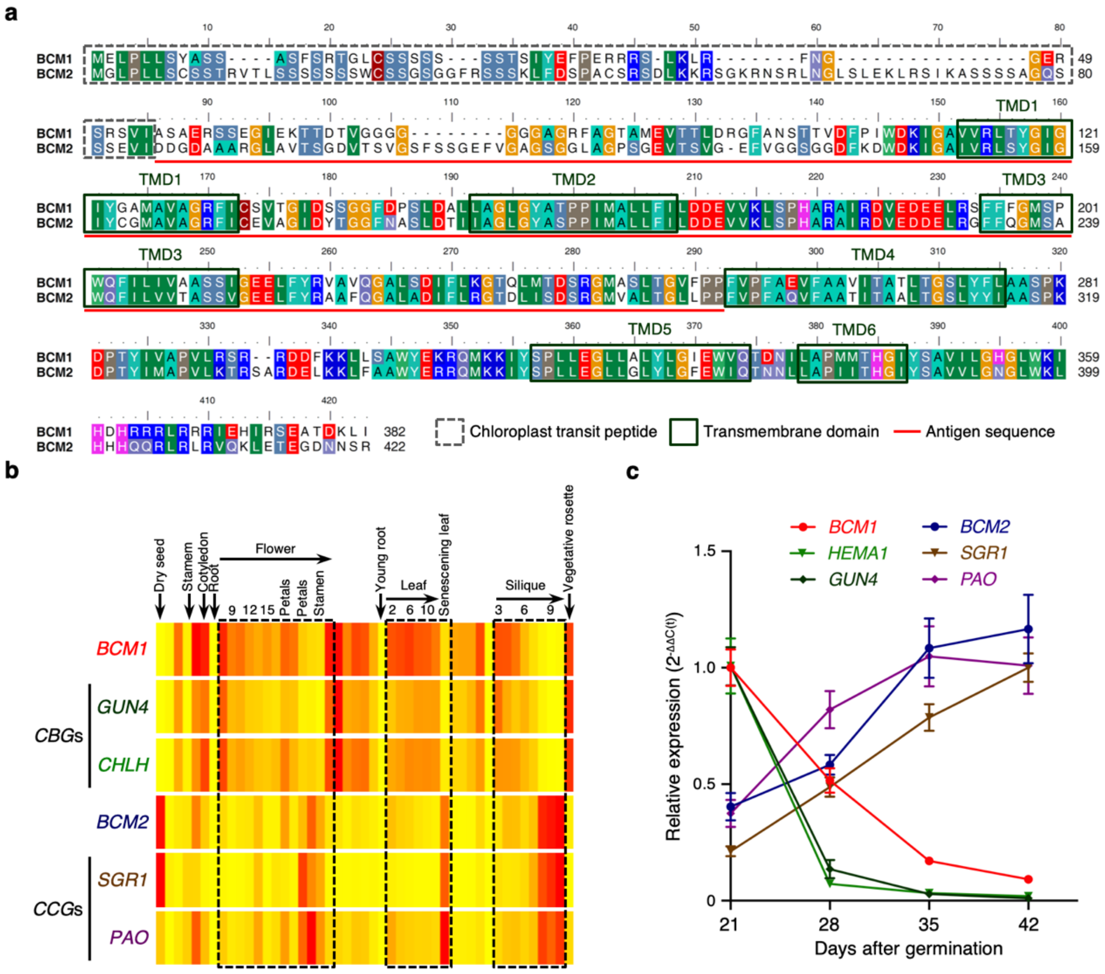

Supplementary Fig. 9. Characterization of BCM paralogs in *Arabidopsis*.

a Protein sequence alignment of *Arabidopsis* BCM1 and BCM2. The chloroplast transit peptide (cTP), transmembrane domain (TMD) and sequence for BCM1 antigen preparation are indicated by gray dotted frame, green box, and red line, respectively. **b** Comparison of the tissue-specific and developmental expression patterns of *BCM1*, *BCM2*, *CGB*s and *CCG*s Data and individual heat maps are from the BAR Expression Angler (http://bar.utoronto.ca/)[1]. The mRNA expression levels are normalized to the maximum expression levels within each gene. Yellow and Red are respectively the lowest and highest expression levels. **c** qRT-PCR analyses of the transcript levels of two *BCMs*, *CBG*s and *CCG*s in 21-, 28-, 35-, and 42-day-old WT seedlings. The relative gene expression levels of *BCM1* and *CBG*s were presented relative to that in 21-day-old WT seedlings. In contrast, the relative transcript levels of *BCM2* and *CCG*s were presented relative to that in 42-day-old WT seedlings. Error bars represent SD of three biological replicates.

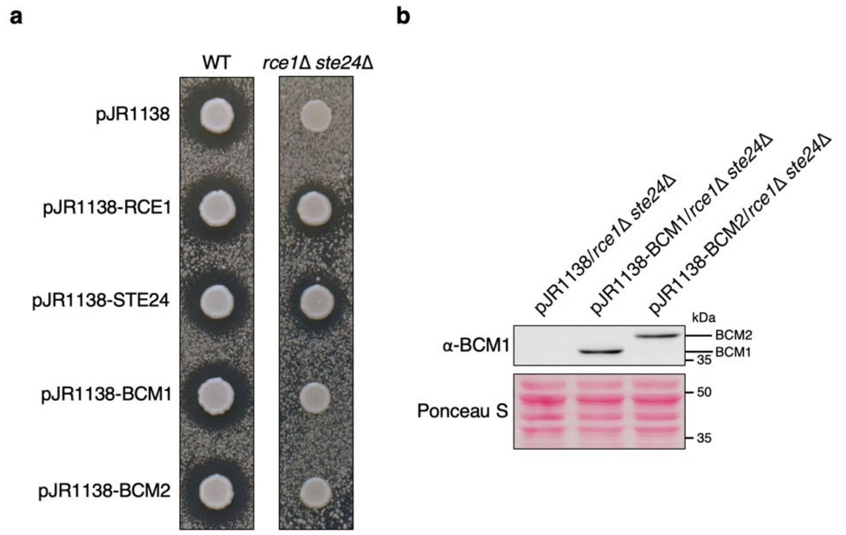

Supplementary Fig. 10. BCM1 and BCM2 cannot restore a-factor secretion in *rce1*Δ *ste241*Δ mutant *S. cerevisiae.*

a Pheromone diffusion (halo) assay was conducted to test **a**-factor processing and secretion. The fully processed **a**-Factor was exported from *MATa* cells, and then arrested the growth of *MATα sst2* strain, forming a zone of growth inhibition (halo). The halo size reflects the amount of **a**-factor produced. Empty vector (pJR1138) was used as a negative control. The plasmids encoding **a**-factor CAAX proteases RCE1 and STE24 were used as positive controls. Representative photograph for yeast halo assay was shown. **b** Expression of *BCM1* and *BCM2* in *rce1*Δ *ste241*Δ mutant *MATa* cells was confirmed by immunoblot analysis using BCM1 antibody. Ponceau S-stained membrane strips bearing total yeast proteins were used as a loading control.

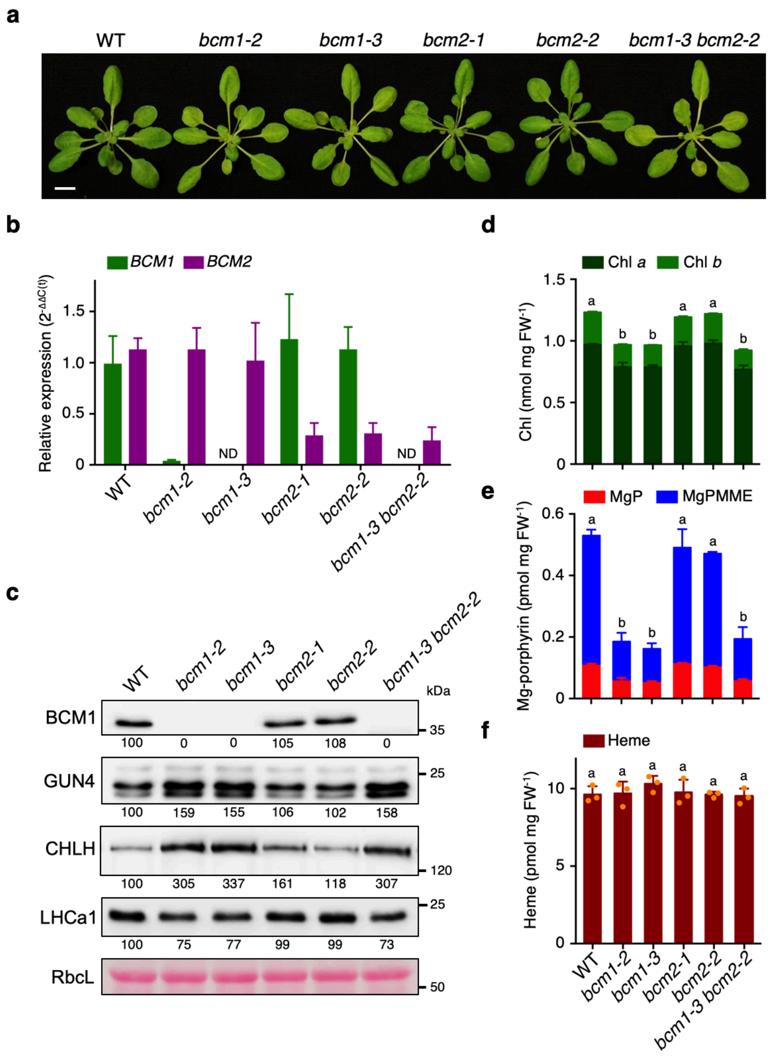

Supplementary Fig. 11. Characterization of *bcm1-3 bcm2-2* double mutant.

a Representative images of 28-day-old WT, *bcm1*, *bcm2* and *bcm1-3 bcm2-2* seedlings grown under short-day normal light (120 μmol photon m^{-2} s^{-1}) conditions. Scale bar, 1 cm. **b** qRT-PCR analysis of *BCM1* and *BCM2* transcripts in the seedlings shown in (**a**). ND, not detected. **c** Steady-state levels of the indicated proteins in the seedlings analyzed in (**a**) were determined by immunoblot analysis using the indicated antibodies. Ponceau S-stained RbcL was used as the loading control. Numbers below immunoblots represent normalized protein abundances relative to WT seedlings. **d**-**f** Levels of Chl (**d**), Mg-porphyrin (**e**) and heme (**f**) in 18-day-old WT, *bcm1*, *bcm2* and *bcm1-3 bcm2-2* seedlings grown under the same conditions as in (**a**). In (**b**) and (**d**)-(**f**), error bars represent SD of three biological replicates.

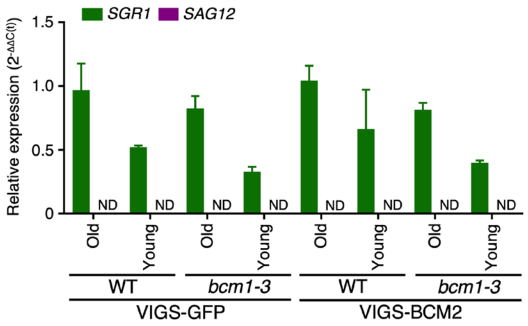

Supplementary Fig. 12. mRNA expression analysis.

qRT-PCR analysis of *SGR1* and *SAG12* transcripts in the old and young mature leaves from 35-day-old VIGS-GFP/WT, VIGS-GFP/*bcm1-3*, VIGS-BCM2/WT, and VIGS-BCM2/*bcm1-3* seedlings shown in Fig. 5c. ND, not detected. Expression levels are presented relative to those in the old leaves of VIGS-GFP/WT seedlings. Error bars represent SD of three biological replicates.

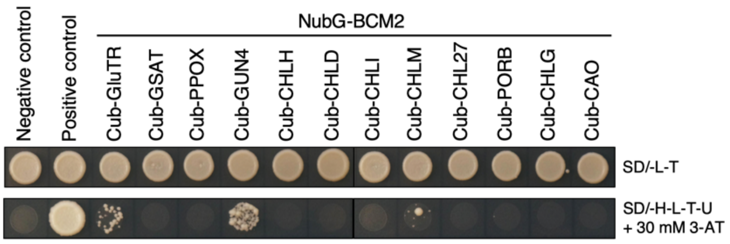

Supplementary Fig. 13. Y2H analyses for interactions between BCM2 and CBEs.
The transformed yeast strains were analyzed on selective medium lacking Leu and Trp (SD/-L-T) or His, Leu, Trp and Ura (SD/-H-L-T-U) in the presence of 30 mM 3-amino-1,2,4-triazole (3-AT). The combination of NubG-GluTR and Cub-GBP was used as the positive control. The NubG was used as the negative control for NubG-BCM1 and NubG-BCM2 in Fig. 4a.

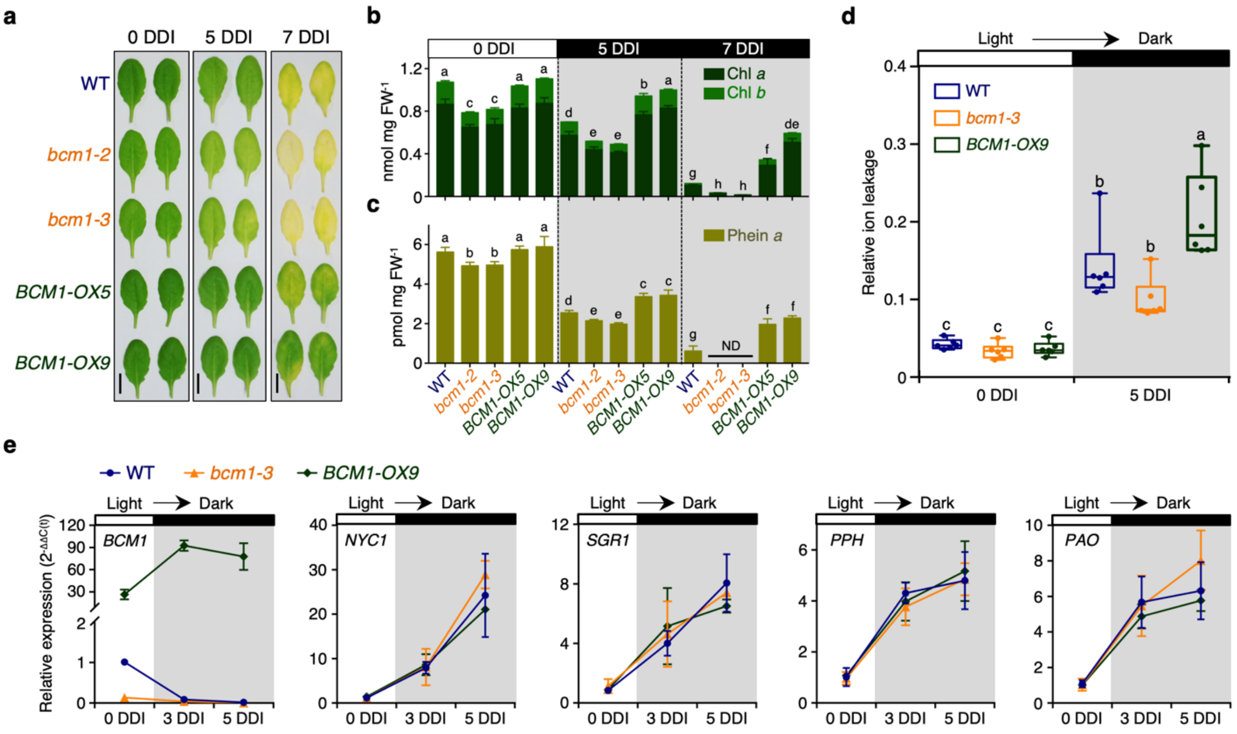

Supplementary Fig. 14. BCM1-OX plants exhibit cosmetic stay-green leaf phenotype during dark incubation.

a Representative images of detached leaves from 35-day-old WT, *bcm1* and *BCM1-OX* seedlings grown under short-day normal light (120 μmol photons m^{-2} s^{-1}) conditions at 0, 5 and 7 DDI. Scale bars, 0.5 cm. **b** and **c** Levels of Chl (**b**) and Phein *a* (**c**) in the detached leaves analyzed in (**a**) at 0, 5, and 7 DDI. ND, not detected. Error bars represent SD of three biological replicates. **d**, Relative ion leakage in WT, *bcm1-3*, and *BCM1-OX9* seedlings was measured at 0 and 5 DDI. Error bars represent SD of five biological replicates. **e** qRT-PCR analysis of *BCM1* and *CCG*s transcripts in the detached leaves analyzed in (**d**). Expression levels are presented relative to those that in WT seedlings at 0 DDI. Error bars represent SD of three biological replicates. In (**b**)-(**d**), letters above histograms indicate significant differences as determined by Tukey's HSD method ($P < 0.05$).

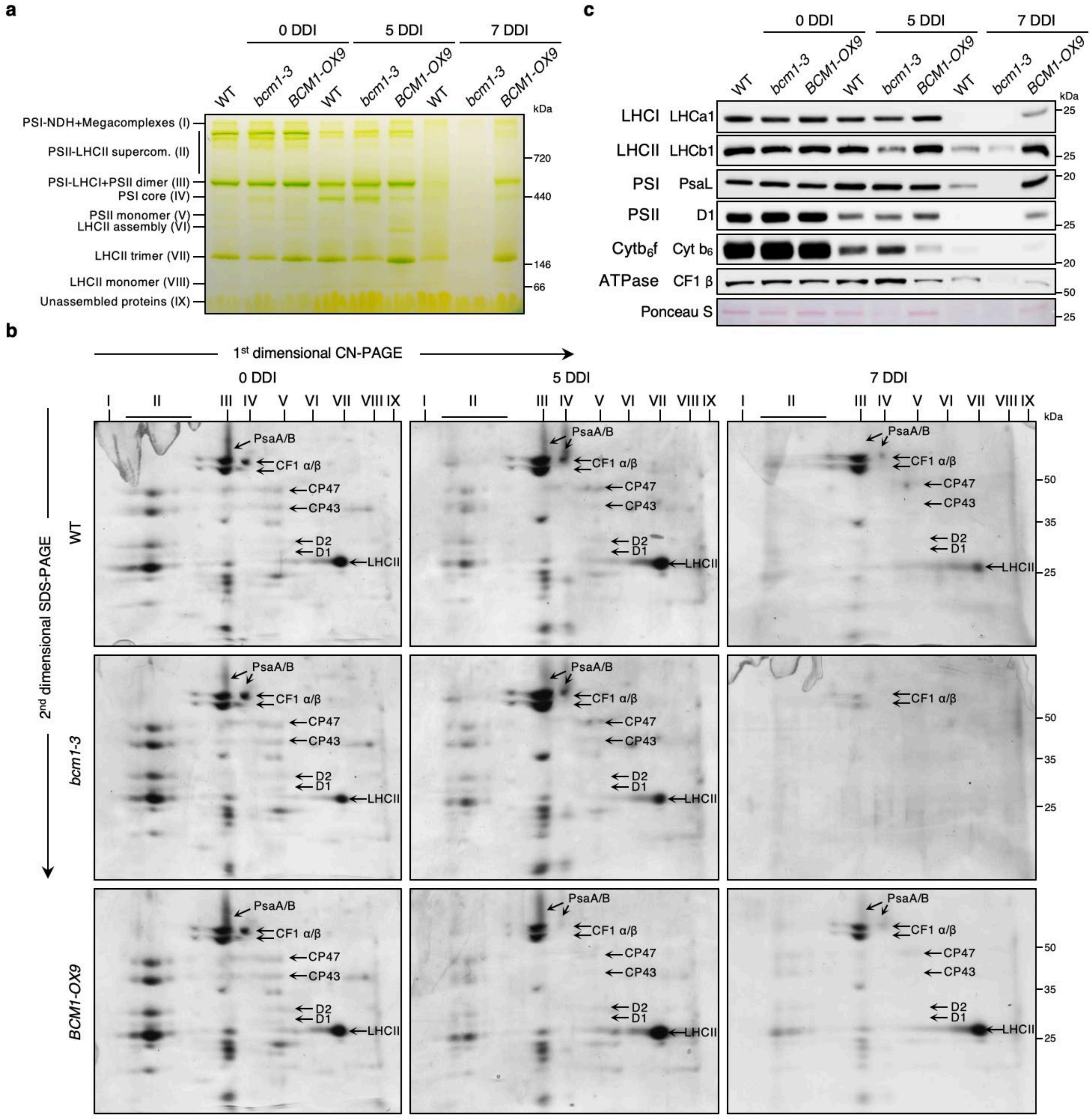

Supplementary Fig. 15. Analyses of thylakoid membrane protein complexes during dark incubation.

a and **b** Two-dimensional clear native (CN)-SDS-PAGE analysis of thylakoid membrane protein complexes from 5-week-old WT, *bcm1-3*, and *BCM1-OX9* seedlings at 0, 5, and 7 DDI. For the first dimensional CN-PAGE analysis in (**a**), equivalent total thylakoid membranes (8 µg of chlorophyll) were solubilized with 1% (w/v) β-DM and separated by 4-12.5% CN-PAGE. The identities of thylakoid protein complexes were assigned as described previously[2]. For the second dimensional SDS-PAGE analyses in (**b**), individual lanes from (**a**)

were subjected to denaturing SDS-PAGE followed by Coomassie Brilliant Blue staining. Identities of the relevant proteins are indicated by arrows. **c** Steady-state levels of thylakoid membrane proteins from 5-week-old WT, *bcm1-3*, and *BCM1-OX9* seedlings at 0, 5, and 7 DDI were detected by immunoblotting using the indicated antibodies. Ponceau S stained total thylakoid proteins were used as loading control. Designations of various thylakoid membrane protein complexes and their components are indicated at left.

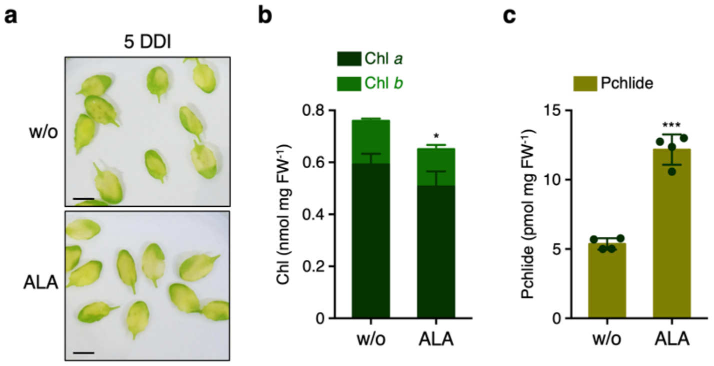

Supplementary Fig. 16. ALA feeding cannot inhibit chlorophyll breakdown during dark incubation.

a Representative images of detached leaves from 3-week-old WT seedlings at 5 DDI. Detached leaves were incubated in PBS buffer without (w/o) or with 0.5 mM ALA. Scale bars, 1 cm. **b** and **c** Levels of Chl (**b**) and Pchlide (**c**) in the detached leaves analyzed in (**a**) at 5 DDI. The more than 2-fold higher Pchlide in ALA samples than that in w/o samples confirmed the functional ALA feeding in this experiment. Error bars represent SD of three biological replicates. Asterisks denote statistically significant differences as determined by a two-tailed Student's *t* tests. **P* < 0.05, ****P* < 0.001.

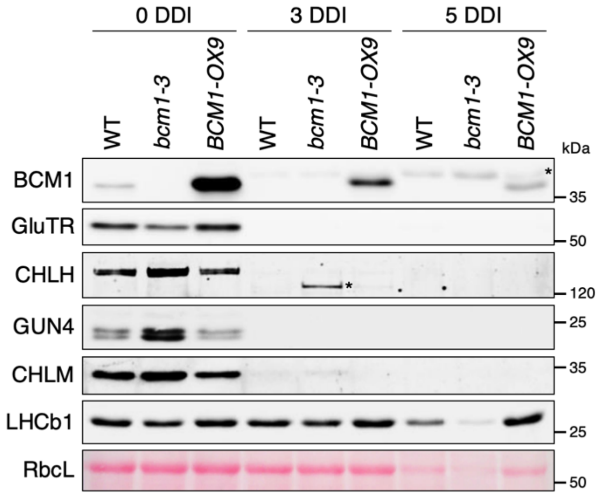

Supplementary Fig. 17. Stability of BCM1, TBS proteins and LHCb1 during dark incubation.

Steady-state levels of indicated proteins in the detached leaves from 35-day-old WT, *bcm1-3* and *BCM1-OX9* seedlings grown under short-day normal light (120 µmol photons m^{-2} s^{-1}) conditions at 0, 3 and 5 DDI were detected by immunoblotting using the indicated antibodies. Ponceau S-stained membrane strips bearing RbcL was used as a loading control. Asterisks indicate nonspecific signals on the immunoblots.

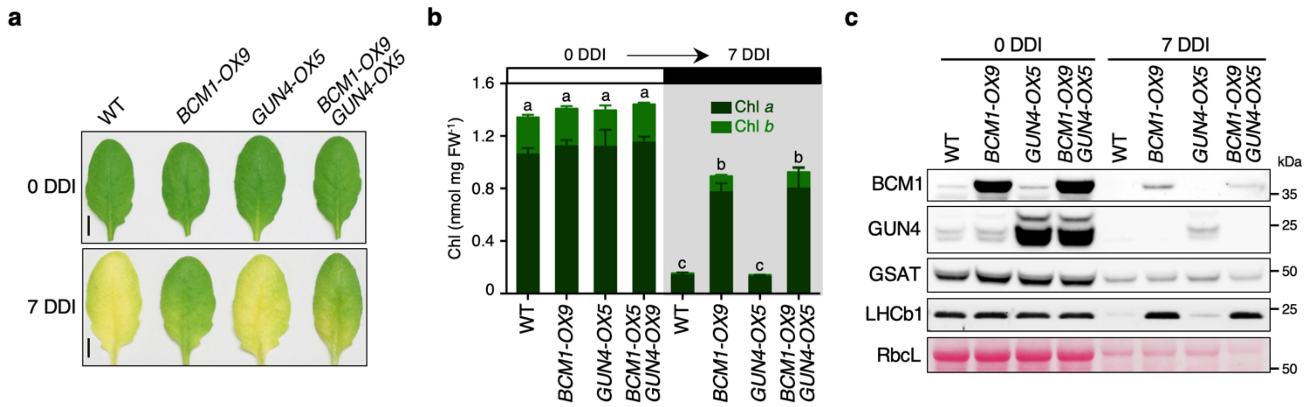

Supplementary Fig. 18. Overexpression of *GUN4* does not lead to a stay-green phenotype during dark incubation.

a Representative images of detached leaves from 35-day-old WT, *BCM1-OX9*, *GUN4-OX5*, *BCM1-OX GUN4-OX5* seedlings grown under short-day normal light (120 µmol photons m^{-2} s^{-1}) conditions at 0 and 7 DDI. Scale bars, 0.5 cm. **b** Levels of Chl in the detached leaves analyzed in (**a**) at 0 and 7 DDI. Error bars represent SD of three biological replicates. Letters above histograms indicate significant differences as determined by Tukey's HSD method ($P < 0.05$). **c** Steady-state levels of indicated proteins in the detached leaves analyzed in (**a**) at 0 and 7 DDI were detected by immunoblotting using the indicated antibodies. Ponceau S-stained membrane strips bearing RbcL was used as a loading control.

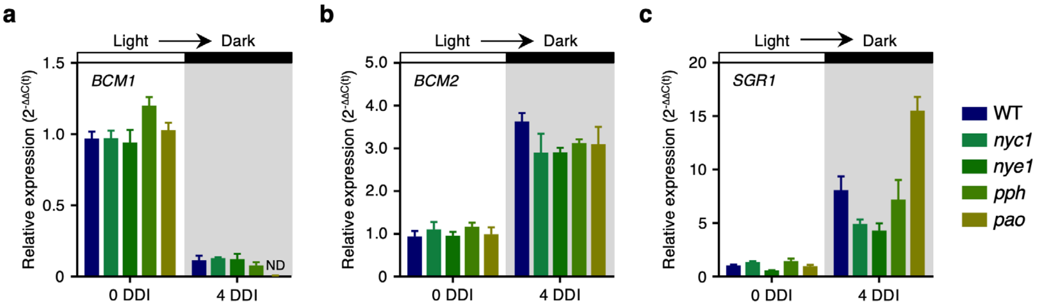

Supplementary Fig. 19. mRNA expression analysis.

a-**c** Gene expression of *BCM1*(**a**), BCM2 (**b**) and *SGR1* (**c**) in the detached leaves from 35-day-old WT, *nyc1*, *nye1*, *pph* and *pao* seedlings grown under short-day normal light (120 μmol photons m^{-2} s^{-1}) conditions at 0 and 4 DDI were determined by qRT-PCR analyses. Expression levels are presented relative to those in WT seedlings at 0 DDI. Error bars represent SD of three biological replicates.

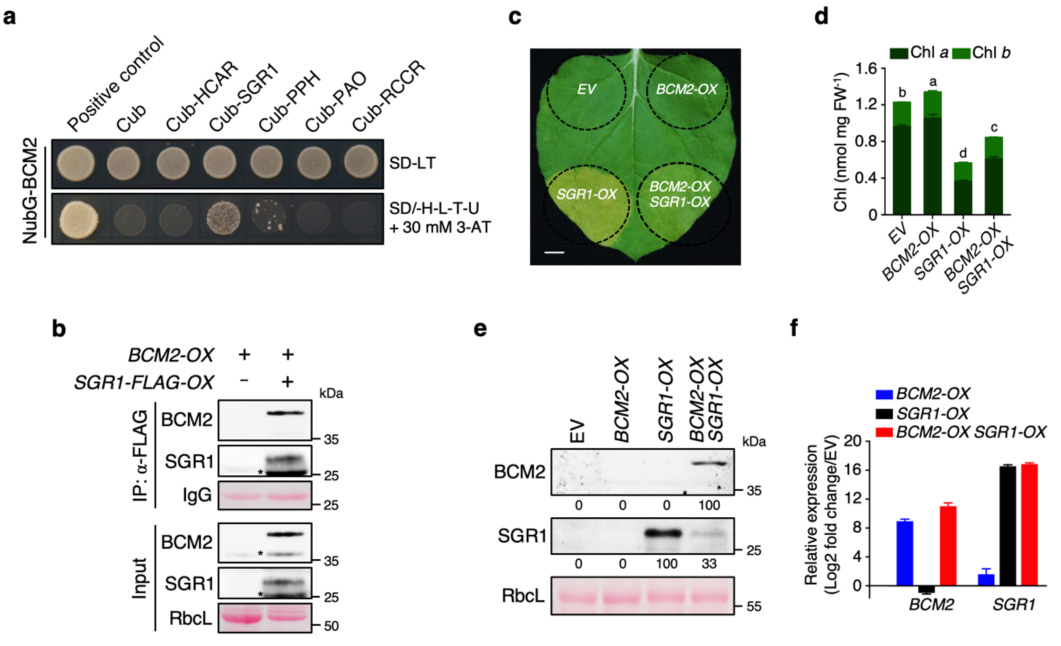

Supplementary Fig. 20. BCM2 interacts with and destabilize SGR1.

a Y2H analyses for interactions between BCM2 and CCEs. The transformed yeast strains were analyzed on selective medium SD/-L-T or SD/-H-U-L-T in the presence of 30 mM 3-AT. The combination of NubG-GluTR and Cub-GBP was used as the positive control. **b** Co-immunoprecipitation experiments demonstrate that BCM2 directly interact with SGR1 in vivo. Anti-FLAG beads were used for immunoprecipitation. Samples of input and precipitated products were analyzed by immunoblot using anti-BCM1 and anti-SGR1 antibodies. **c** Representative image of a tobacco leaf with zones overexpressing the empty vector (EV), *BCM2*, *SGR1* and both *BCM2* and *SGR1* after 2 days of growth in dark. The infiltrated leaf areas are indicated by circles. Scale bar, 1 cm. **d** Levels of Chl Levels of Chl in the infiltrated leaf areas in (**c**). Error bars represent SD of three biological replicates. Letters above bars indicate significant differences determined by Tukey's HSD method ($P < 0.05$). **e** Steady-state levels of BCM2 and SGR1 in the infiltrated leaf areas in (**c**) were detected by immunoblotting using the indicated antibodies. Numbers below the immunoblot image represent normalized protein abundance in the examined genotypes relative to EV seedlings. **f** qRT-PCR analysis of *BCM2* and *SGR1* transcripts, confirming overexpression of these genes in the infiltrated leaf areas in (**c**). Expression levels are presented relative to those in EV. In (**b**) and (**f**), Ponceau S-stained membrane strips bearing RbcL or the light chain of IgG were used as loading controls.

Supplementary Table 1. ***Arabidopsis*** **mutant lines used in this study.**

Mutant	Gene	Description	Mutation	Reference
bcm1-1	*BCM1*/*At*2g35260	*balance of chlorophyll metabolism 1*	Salk_112780, T-DNA in 5' UTR	This study
bcm1-2	*BCM1*/*At*2g35260	*balance of chlorophyll metabolism 1*	Salk_031802, T-DNA in exon 1	This study
bcm1-3	*BCM1*/*At*2g35260	*balance of chlorophyll metabolism 1*	Salk_058830, T-DNA in exon 6	This study
bcm2-1	*BCM2*/*At*4g17840	*balance of chlorophyll metabolism 2*	Salk_010312, T-DNA in 5' UTR	This study
bcm2-2	*BCM2*/*At*4g17840	*balance of chlorophyll metabolism 2*	Salk_138694, T-DNA in 5' UTR	This study
gun5-1	*CHLH*/*At*5g13630	*genomes uncoupled 5-1*	Point mutation, A990V	3
cch	*CHLH*/*At*5g13630	*conditional chlorina*	Point mutation, P642L	3
gun4-1	*GUN4*/*At*3g59400	*genomes uncoupled 4-1*	Point mutation, L88F	4
gun4-3	*GUN4*/*At*3g59400	*genomes uncoupled 4-3*	Salk_011461, T-DNA in 3' border of exon	5
chl27	*CHL27*/*At*3g56940	*Mg‑protophorphyrin monomelthylester cyclase*	Salk_009052, T-DNA in 5' UTR	6
ch1-2	*CAO*/*At*1g44446	*chlorina 1-2*	Point mutantion, V274E	7
nyc1	*NYC1*/*At*4g13250	*non-yellowing coloring 1*	Salk_091664, T-DNA in 3' UTR	8
nye1	*NYE1*/*SGR1* /*At*4g22920	*non-yellowing 1/stay-green 1*	Nonsense mutation at L10	9
pph	*PPH*/*At*5g13800	*pheophytinase*	Salk_000095, T-DNA in exon 3	10
pao	*PAO*/*At*3g44880	*pheophorbide a oxygense*	Salk_111333, T-DNA in intron 5	11
bcm1-3 bcm2-2		Double mutant of *bcm1-3* and *bcm2-2*		This study
bcm1-3 gun5-1		Double mutant of *bcm1-3* and *gun5-1*		This study
bcm1-3 gun4-1		Double mutant of *bcm1-3* and *gun4-1*		This study
bcm1-3 gun4-3		Double mutant of *bcm1-3* and *gun4-3*		This study
bcm1-3 chl27		Double mutant of *bcm1-3* and *chl27*		This study

Supplementary Table 2. Primers used for genotyping

Primer	Sequence (5'-3')	Purposes
LBa1	TGGTTCACGTAGTGGGCCATCG	T-DNA left border primer 1
SALK_112780-LP	TCCACCAGATGAATCAATTCC	Genotyping of *bcm1-1*
SALK_112780-RP	ACAACATAAATGAAAACCATTGTC	Genotyping of *bcm1-1*
SALK_031802- LP	TCAAAATAACCACCAAAGTTTATG	Genotyping of *bcm1-2*
SALK_031802- RP	TCCACCAGATGAATCAATTCC	Genotyping of *bcm1-2*
SALK_058830- LP	AAGGCCCAATGGAAATTATTG	Genotyping of *bcm1-3*
SALK_058830- RP	TTTGTCCCATTTGCTGAAGTC	Genotyping of *bcm1-3*
SALK_010312- LP	GATGCATGTAGGTCGAATTGC	Genotyping of *bcm2-1*
SALK_010312- RP	TTCAAATCCGATCTCGAACAC	Genotyping of *bcm2-1*
SALK_138694- LP	GATGCATGTAGGTCGAATTGC	Genotyping of *bcm2-2*
SALK_138694- RP	TTCAAATCCGATCTCGAACAC	Genotyping of *bcm2-2*
SALK_091664- LP	TGGACTTAGGCAGTTTCATGG	Genotyping of *nyc1*
SALK_091664- RP	TAAAAAGCCTATTTGCCGACC	Genotyping of *nyc1*
SALK_000095- LP	CTACCAATCCTGGACTCCTCC	Genotyping of *pph*
SALK_000095- RP	TGTACAGGTTATCGGTGAGCC	Genotyping of *pph*
SALK_111333- LP	GAAAATGGTTGGGATAGAGCC	Genotyping of *pao*
SALK_111333- RP	TGTAAGCTCCTTTGCAGGAAG	Genotyping of *pao*

Supplementary Table 3. Primers used for plasmid construction

Primer	Sequence (5'-3')	Purposes
pGL1-BCM1-Fw	CCCGGGATGGAGCTTCCGTTACT	Plant transformation
pGL1-BCM1-Rev	CCCGGGTTAAATCAACTTATCCGTGG	Plant transformation
pGL1-BCM2-Fw	TCTAGAATGGGTCTTCCTTTATTGT	Plant transformation
pGL1-BCM2-Rev	CCCGGGCTATCTTGAGTTGTTGTCAC	Plant transformation
pGL1-GUN4-Fw	TCTAGAATGGCGACCA CAAACTCTC	Plant transformation
pGL1-GUN4-Rev	CCCGGGTCAGAAGCTGTAATTTGT	Plant transformation
pGL1-SGR1-FLAG-Fw	ACCCGGGATGTGTAGTTTGTCGGCG	Plant transformation
pGL1-SGR1-FLAG-Rev	TCCCGGGCTACTTGTCATCATCGTCC TTGTAGTCGAGTTTCTCCGGATT	Plant transformation
VIGS-BCM2-Fw	AAGGTACCTAGTGGCGGTTTAGCT	VIGS assay
VIGS-BCM2-Rev	TTACGCGTGCTGCAAACAACTTCT	VIGS assay
pUC-BCM1-Fw	GGATCCATGGAGCTTCCGTTACTCT	Subcellular localization analysis
pUC-BCM1-Rev	CTCGAGAATCAACTTATCCGTGGCC	Subcellular localization analysis
pUC-cTP$_{BCM1}$-Fw	AAGGATCCATGGAGCTTCCGTTACT	Subcellular localization analysis
pUC-cTP$_{BCM1}$-Rev	CACTCGAGGACGGTGTCTGTTGTCT	Subcellular localization analysis
pJR1138-BCM1-Fw	GGATCCCCATGGCTTCCGCTGAAC	Yeast complementation assay
pJR1138-BCM1-Rev	GGATCCAATCAACTTATCCGTGGCC	Yeast complementation assay
pJR1138-BCM2-Fw	GGATCCCCATGGCATCGTCGTCTTCC	Yeast complementation assay
pJR1138-BCM2-Rev	GGATCCTCTTGAGTTGTTGTCACCT	Yeast complementation assay
pJR1138-RCE1-Fw	GGATCCCCATGGCCACCGATGGCG	Yeast complementation assay
pJR1138-RCE1-Rev	GAGCTCGATTCCACAAACAATAGCCAAG	Yeast complementation assay
pJR1138-STE24-Fw	GGATCCCCATGGCGATTCCTTTCATGG	Yeast complementation assay
pJR1138-STE24-Rev	GGATCCATCTGTCTTCTTGTCTTCTC	Yeast complementation assay
pXNgate-BCM1-Fw	CAAAAAAGCAGGCTTAATGGCTTCCGCT GAACGGAGCAG	Split-ubiquitin membrane-based Y2H
pXNgate-BCM1-Rev	CAAGAAAGCTGGGTGTCAAATCAACTTA TCCGTGGCCT	Split-ubiquitin membrane-based Y2H
pXNgate-BCM2-Fw	CAAAAAAGCAGGCTTAATGGCATCGTCG TCTTCCGCTGG	Split-ubiquitin membrane-based Y2H
pXNgate-BCM2-Rev	CAAGAAAGCTGGGTGTCATCTTGAGTTG TTGTCACCTT	Split-ubiquitin membrane-based Y2H
pDHB1-BCM1-Fw	CCTAGGGCTTCCGCTGAACGGAGC	Split-ubiquitin membrane-based Y2H
pDHB1-BCM1-Rw	CTGCAGAATCAACTTATCCGTGGCC	Split-ubiquitin membrane-based Y2H
pDHB1-GUN4-Fw	CCTAGGGCCTCCGCCACAACTGCCG	Split-ubiquitin membrane-based Y2H
pDHB1-GUN4-Rev	CTGCAGGAAGCTGTAATTTGTTTT	Split-ubiquitin membrane-based Y2H
pDHB1-CHLH-Fw	CCTAGGGCTCAGTACCAGTCTTCTC	Split-ubiquitin membrane-based Y2H
pDHB1-CHLH-Rev	GCTAGCTCGATCGATCCCTTCGATC	Split-ubiquitin membrane-based Y2H

pDHB1-HCAR-Fw	GCCTAGGTCTTCTTCTTCGCGTTCC	Split-ubiquitin membrane-based Y2H
pDHB1-HCAR-Rev	TCTGCAGCTTGTCATCATCGTCCTTGTAGTCTTT CTTGGAGAGCAT	Split-ubiquitin membrane-based Y2H
pDHB1-SGR1-Fw	GACTAGTGCAAGGTTGTTTGGACC	Split-ubiquitin membrane-based Y2H
pDHB1-SGR1-Rev	TCTGCAGCTTGTCATCATCGTCCTTGTAGTCGAG TTTCTCCGGATT	Split-ubiquitin membrane-based Y2H
pDHB1-PPH-Fw	GCCTAGGAGTGGAAATTCCGATGGT	Split-ubiquitin membrane-based Y2H
pDHB1-PPH-Rev	TACTAGTCTTGTCATCATCGTCCTTGTAGTCTGCA GACTTCCCTCC	Split-ubiquitin membrane-based Y2H
pDHB1-PAO-Fw	GACTAGTGTGGCGGCGCCGCCGTCT	Split-ubiquitin membrane-based Y2H
pDHB1-PAO-Rev	TCTGCAGCTTGTCATCATCGTCCTTGTAGTCCTCG ATTTCAGAATG	Split-ubiquitin membrane-based Y2H
pDHB1-RCCR-Fw	GCCTAGGTCCATGGAAGACCACGAC	Split-ubiquitin membrane-based Y2H
pDHB1-RCCR-Rev	TCTGCAGCTTGTCATCATCGTCCTTGTAGTCGAGA ACACCGAAAGC	Split-ubiquitin membrane-based Y2H
pDonor-BCM1-Fw	CAAAAAAGCAGGCTGAATGATGGAGCTTCCGTTA CTCTC	BiFC
pDonor-BCM1$^{\Delta C20}$-Rev	CAAGAAAGCTGGGTGGTGATCGTGAATTTTCCAC AGCC	BiFC
pDonor-GUN4-Fw	CAAAAAAGCAGGCTGAATGATGGCGACCA CAAAC	BiFC
pDonor-GUN4-Rev	CAAGAAAGCTGGGTG GAAGCTGTAATTTGTTTT	BiFC
pDonor-SGR1-Fw	CAAAAAAGCAGGCTGAATGTGTAGTTTGTCGGCG	BiFC
pDonor-SGR1-Rev	CAAGAAAGCTGGGTGGAGTTTCTCCGGATT	BiFC
pDonor-PPH-Fw	CAAAAAAGCAGGCTGAATGGAGATAATCTCACTG	BiFC
pDonor-PPH-Rev	CAAGAAAGCTGGGTGTGCAGACTTCCCTCC	BiFC
pDonor-PAO-Fw	CAAAAAAGCAGGCTGAATGTCAGTAGTTTTACTC	BiFC
pDonor-PAO-Rev	CAAGAAAGCTGGGTGCTCGATTTCAGAATGT	BiFC
pET28-BCM1$^{55\text{-}253}$-Fw	CATATGGCTTCCGCTGAACGGAGC	Expression of BCM1 antigen in *E. coli*
pET28-BCM1$^{55\text{-}253}$-Rev	CTCGAGTGGAGGAAACACTCCGGTC	Expression of BCM1 antigen in *E. coli*
pDR296-BCM1-Fw	AACCCGGGATGGGCAGCAGCCATCAT	Expression of BCM1 in *Saccharomyces cerevisiae*
pDR296-BCM1-Rev	AACTCGAGTTAAATCAACTTATCCGT	Expression of BCM1 in *Saccharomyces cerevisiae*

Supplementary Table 4. Primers used for gene expression analyses

Primer	Sequence (5'-3')	Purposes
UBQ10-Fw	CCCTTCATCTTGTTCTCAG	RT-PCR
UBQ10-Rev	CAGCCAAAGTTCTTCCAT	RT-PCR
BCM1-Fw	ATGGAGCTTCCGTTACTCTCGTATG	RT-PCR
BCM1-Rev	TTAAATCAACTTATCCGTGGCCTCC	RT-PCR
BCM2-Fw	ATCAAAATCAATGATCAAGGTAACG	RT-PCR
BCM2-Rev	AACCGACGTTACATCTCCTGAAGTA	RT-PCR
SAND-Fw	AACTCTATGCAGCATTTGATCCACT	qRT-PCR
SAND-Rev	TGATTGCATATCTTTATCGCCATC	qRT-PCR
Nt-α-TUBULIN-Fw	CAAGACTAAGCGTACCATCCA	qRT-PCR
Nt-α-TUBULIN-Fw	TTGAATCCAGTAGGGCACCAG	qRT-PCR
BCM1-Fw	CTCTACTTTCTTGCTGCGTCTC	qRT-PCR
BCM1-Rev	GCGTCATCATCGGTGCTA	qRT-PCR
BCM2-Fw	GGGTCTTCCTTTATTGTCTTGT	qRT-PCR
BCM2-Rev	TCTCCGTCGTCGATCACTT	qRT-PCR
HEMA1-Fw	TTGCTGCCAACAAAGAAGAC	qRT-PCR
HEMA1-Rev	CCGTCTCCAATGAATCCCTC	qRT-PCR
GSAT1-Fw	TCAAAGAAGAGCGACACAGAG	qRT-PCR
GSAT1-Rev	GTAAACACCTTCTTCCAACATTCC	qRT-PCR
GUN4-Fw	TGATGGTAGATTCGGATACAGC	qRT-PCR
GUN4-Rev	CAAGAAGCTTCATCCACTCAAC	qRT-PCR
CHLH-Fw	CTGGTCGTGACCCTAGAACAG	qRT-PCR
CHLH-Rev	GATTGCCAGCTTCTTCTCTG	qRT-PCR
CHLM-Fw	TTGCTGAAGCTGAGATGAAGGCA	qRT-PCR
CHLM-Rev	CAACGGTATCATACTTCCCAGTTAG	qRT-PCR
CHL27-Fw	GCTTCTTCTGCCTCTCGGTTTATG	qRT-PCR
CHL27-Rev	GCCGTGGTTCGGTTTGTCTCG	qRT-PCR
PORB-Fw	TGATTACCCTTCAAAGCGTCTCA	qRT-PCR
PORB-Rev	CAATGTATTCGTGTTCCCGGT	qRT-PCR
CHLG-Fw	TCATTCCTCAGATTGTGTTCCA	qRT-PCR
CHLG-Rev	GTTACAAATATTCCGAGCACCA	qRT-PCR
CAO-Fw	AAGGCTGGAGTGTCCCAAGT	qRT-PCR
CAO-Rev	ATCCTTGGAGACCCGAGGTAG	qRT-PCR
LHCA1-Fw	AAGTATGGAGAAAGACCCTGAG	qRT-PCR
LHCA1-Rev	GAATCCTACAAACGCCAACAG	qRT-PCR
LHCB2.1-Fw	TCATTTGGCTGATCCTGTGG	qRT-PCR
LHCB2.1-Rev	GTACATTCACGACTTTACAAAGCAG	qRT-PCR
NYC1-Fw	ACTTCTTCTCAGTGGTTCGAG	qRT-PCR
NYC1-Rev	AGGAGGAGTTAGGTAATTGACGG	qRT-PCR
SGR1-Fw	GCTGTTTCGCCTGATGG	qRT-PCR
SGR1-Rev	TTTGGAGTAGCAATTCCCTC	qRT-PCR
PPH-Fw	CTTTGGCGTTGGTTCATTT	qRT-PCR
PPH-Rev	CAGCCCACGGTTCAGTTT	qRT-PCR
PAO-Fw	TCACTCCAACCCAGGCAGAC	qRT-PCR

PAO-Rev	GATAAACCAGCAAGAACCAGTCG	qRT-PCR
SAG12-Fw	AAAGGAGCTGTGACCCCTATCAA	qRT-PCR
SAG12-Rev	CCAACAACATCCGCAGCTG	qRT-PCR

Supplementary References

1 Waese, J. *et al.* ePlant: Visualizing and Exploring Multiple Levels of Data for Hypothesis Generation in Plant Biology. *The Plant cell* **29**, 1806-1821, doi:10.1105/tpc.17.00073 (2017).

2 Jarvi, S., Suorsa, M., Paakkarinen, V. & Aro, E. M. Optimized native gel systems for separation of thylakoid protein complexes: novel super- and mega-complexes. *Biochem J* **439**, 207-214, doi:10.1042/BJ20102155BJ20102155 [pii] (2011).

3 Mochizuki, N., Brusslan, J. A., Larkin, R., Nagatani, A. & Chory, J. Arabidopsis genomes uncoupled 5 (GUN5) mutant reveals the involvement of Mg-chelatase H subunit in plastid-to-nucleus signal transduction. *Proceedings of the National Academy of Sciences of the United States of America* **98**, 2053-2058, doi:10.1073/pnas.98.4.2053 (2001).

4 Larkin, R. M., Alonso, J. M., Ecker, J. R. & Chory, J. GUN4, a regulator of chlorophyll synthesis and intracellular signaling. *Science* **299**, 902-906, doi:10.1126/science.1079978 (2003).

5 Richter, A. S. *et al.* Phosphorylation of GENOMES UNCOUPLED 4 Alters Stimulation of Mg Chelatase Activity in Angiosperms. *Plant physiology* **172**, 1578-1595, doi:10.1104/pp.16.01036 (2016).

6 Bang, W. Y. *et al.* Role of Arabidopsis CHL27 protein for photosynthesis, chloroplast development and gene expression profiling. *Plant Cell Physiol* **49**, 1350-1363, doi:10.1093/pcp/pcn111 (2008).

7 Wang, P. & Grimm, B. Comparative Analysis of Light-Harvesting Antennae and State Transition in chlorina and cpSRP Mutants. *Plant physiology* **172**, 1519-1531, doi:10.1104/pp.16.01009 (2016).

8 Horie, Y., Ito, H., Kusaba, M., Tanaka, R. & Tanaka, A. Participation of chlorophyll b reductase in the initial step of the degradation of light-harvesting chlorophyll a/b-protein complexes in Arabidopsis. *The Journal of biological chemistry* **284**, 17449-17456, doi:10.1074/jbc.M109.008912 (2009).

9 Ren, G. *et al.* Identification of a novel chloroplast protein AtNYE1 regulating chlorophyll degradation during leaf senescence in Arabidopsis. *Plant physiology* **144**, 1429-1441, doi:10.1104/pp.107.100172 (2007).

10 Schelbert, S. *et al.* Pheophytin pheophorbide hydrolase (pheophytinase) is involved in chlorophyll breakdown during leaf senescence in Arabidopsis. *The Plant cell* **21**, 767-785, doi:10.1105/tpc.108.064089 (2009).

11 Pruzinska, A. *et al.* Chlorophyll breakdown in senescent Arabidopsis leaves. Characterization of chlorophyll catabolites and of chlorophyll catabolic enzymes involved in the degreening reaction. *Plant physiology* **139**, 52-63, doi:10.1104/pp.105.065870 (2005).
