# Supplementary material for: Post-translational coordination of chlorophyll biosynthesis and breakdown by BCMs maintains chlorophyll homeostasis during leaf development
Source: Nat Commun. 2020 Mar 20;11:1254. doi: 10.1038/s41467-020-14992-9 (PMC7083845; doi:10.1038/s41467-020-14992-9)
Supplement: Supplementary file 3 — Reporting Summary [file 41467_2020_14992_MOESM3_ESM.pdf]

nature research

Corresponding author(s): Peng Wang, Bernhard Grimm

Last updated by author(s): Feb 7, 2020

Reporting Summary

Nature Research wishes to improve the reproducibility of the work that we publish. This form provides structure for consistency and transparency in reporting. For further information on Nature Research policies, see Authors & Referees and the Editorial Policy Checklist.

Statistics

For all statistical analyses, confirm that the following items are present in the figure legend, table legend, main text, or Methods section.

n/a	Confirmed	
☐	☒	The exact sample size (*n*) for each experimental group/condition, given as a discrete number and unit of measurement
☐	☒	A statement on whether measurements were taken from distinct samples or whether the same sample was measured repeatedly
☐	☒	The statistical test(s) used AND whether they are one- or two-sided<br>*Only common tests should be described solely by name; describe more complex techniques in the Methods section.*
☒	☐	A description of all covariates tested
☐	☒	A description of any assumptions or corrections, such as tests of normality and adjustment for multiple comparisons
☐	☒	A full description of the statistical parameters including central tendency (e.g. means) or other basic estimates (e.g. regression coefficient) AND variation (e.g. standard deviation) or associated estimates of uncertainty (e.g. confidence intervals)
☐	☒	For null hypothesis testing, the test statistic (e.g. *F*, *t*, *r*) with confidence intervals, effect sizes, degrees of freedom and *P* value noted<br>*Give P values as exact values whenever suitable.*
☒	☐	For Bayesian analysis, information on the choice of priors and Markov chain Monte Carlo settings
☒	☐	For hierarchical and complex designs, identification of the appropriate level for tests and full reporting of outcomes
☒	☐	Estimates of effect sizes (e.g. Cohen's *d*, Pearson's *r*), indicating how they were calculated

Our web collection on statistics for biologists contains articles on many of the points above.

Software and code

Policy information about availability of computer code

Data collection	ChemStation for LC 3D systems was used in HPLC analyses.<br>Leica Application Suite AF (version 2.7.9723.3) was used to capture fluorescent signals.<br>Gatan DigitalMicrograph software (version1.85.1535) was used to capture transmission electron microscopy image.<br>Biorad CFX-manager software (version 1.6) was used in qRT-PCR analyses.<br>ChemoStar Touch (version 0.5.61) was used inImmunoblot analyses.
Data analysis	Co-expression analyses were performed using ATTEDII (http://atted.jp/).<br>Subcellular localization analyses were performed using Target 1.1 Server (http://www.cbs.dtu.dk/services/TargetP/).<br>Prediction of transmembrane domains were performed using TMHMM Server V. 2.0 (http://www.cbs.dtu.dk/services/TMHMM/).<br>Quantification of Western blot results was performed using ImageJ software (US National Institutes of Health).<br>Graphing and statistic analyses were performed using GraphPad Prism 7.0 (https://www.graphpad.com/scientific-software/prism/).<br>A Neighbor-joining tree was constructed in MEGA X (https://www.megasoftware.net/).

For manuscripts utilizing custom algorithms or software that are central to the research but not yet described in published literature, software must be made available to editors/reviewers. We strongly encourage code deposition in a community repository (e.g. GitHub). See the Nature Research guidelines for submitting code & software for further information.

Data

Policy information about availability of data

All manuscripts must include a data availability statement. This statement should provide the following information, where applicable:

- Accession codes, unique identifiers, or web links for publicly available datasets
- A list of figures that have associated raw data
- A description of any restrictions on data availability

All data involved in this study are available from the corresponding authors upon reasonable request.

Field-specific reporting

Please select the one below that is the best fit for your research. If you are not sure, read the appropriate sections before making your selection.

- [x] Life sciences
- [ ] Behavioural & social sciences
- [ ] Ecological, evolutionary & environmental sciences

For a reference copy of the document with all sections, see nature.com/documents/nr-reporting-summary-flat.pdf

Life sciences study design

All studies must disclose on these points even when the disclosure is negative.

Sample size	The sample size in this study is mainly determined according to the prior experiences, which are based on the reproducibility and statistical significance of the results during the experiments.
Data exclusions	No data were excluded from our analyses.
Replication	The number of replication are indicated in the figure legends. All experiments were conducted at least three replicates. All data presented are reliable and reproducible.
Randomization	All samples were arranged randomly into experimental groups.
Blinding	All the experiments were performed without prior knowledge of the final outcome, and therefore blinding was not applied.

Reporting for specific materials, systems and methods

We require information from authors about some types of materials, experimental systems and methods used in many studies. Here, indicate whether each material, system or method listed is relevant to your study. If you are not sure if a list item applies to your research, read the appropriate section before selecting a response.

Materials & experimental systems

n/a	Involved in the study	
☐	☒	Antibodies
☒	☐	Eukaryotic cell lines
☒	☐	Palaeontology
☒	☐	Animals and other organisms
☒	☐	Human research participants
☒	☐	Clinical data

Methods

n/a	Involved in the study	
☒	☐	ChIP-seq
☒	☐	Flow cytometry
☒	☐	MRI-based neuroimaging

Antibodies

Antibodies used

In immunoblot analyses, the following antibodies were used:
Antibody against CHLH (dilution: 1:1000) was provided by Dr. Da-Peng Zhang (Tsinghua University, China). Antibody against CHLI (dilution: 1:5000) was provided by Dr. Meizhong Luo (Huazhong Agricultural University, China). Antibody against SGR1 (dilution: 1:500) was provided by Dr. Ayumi Tanaka (Hokkaido University, Japan).
Antibodies against BCM1 (dilution: 1:500), GluTR (dilution: 1:1000), GSAT (dilution: 1:2000), GUN4 (dilution: 1:2000), CHLM (dilution: 1:500) were prepared in Dr. Bernhard Grimm's lab and described previously (as proved below).
Antibodies against CHL27 (AS06122, dilution: 1:1000), D1 (AS05084, dilution: 1:5000), PsaL (AS06108, dilution: 1:2500), LHCa1 (AS01005, dilution: 1:2500), LHCb1 (AS09522, dilution: 1:2500), Cyt b6 (AS184169, dilution: 1:2500), CF1 β (AS05085, dilution: 1:5000), Tic40 (AS10709, dilution: 1:2500) were purchased from Agrisera.
In co-immunoprecipitation analyses, anti-FLAG affinity gel (B23101) was purchased from Bimake.

Validation

The specificity of antibody in Arabidopsis against CHLH was verified in previous publication: Shen YY, Wang XF, Wu FQ, Du SY, Cao Z, et al. (2006). Nature. 443:823-826.
The specificity of antibody in Arabidopsis against CHLI was verified in previous publication: Luo T, Fan T, Liu Y, Rothbart M, Yu J, et al. (2012). Plant Physiol. 159:118-130.
The specificity of antibodies in Arabidopsis against GluTR and GSAT were verified in previous publication: Czarnecki O, Hedtke B, Melzer M, Rothbart M, Richter A, et al. (2011). Plant Cell. 23:4476-4491.
The specificity of antibodies in Arabidopsis against GUN4 and CHLM were verified in previous publication: Richter AS, Hochheuser C, Fufezan C, Heinze L, Kuhnert F, et al. (2016). Plant Physiol 172:1578-1595.
The specificity of antibodies in Arabidopsis against SGR1 and BCM1 were verified by immunoblot analyses in this study.
The specificity of all commercially obtained antibodies has been validated by the manufacturers (Agrisera, or Bimake):
Validation statement for anti-CHL27 can be found at the product website < https://www.agrisera.com/en/artiklar/crd1-cth1-copper-response-defect-1-chl27marker-of-envelopes-and-thylakoid-membrane.html>.
Validation statement for anti-D1 can be found at the product website < https://www.agrisera.com/en/artiklar/psba-d1-protein-

of-psii-c-terminal-100-l.html>.
Validation statement for anti-PsaL can be found at the product website < https://www.agrisera.com/en/artiklar/psal-psi-l-subunit-of-photosystem-i.html>.
Validation statement for anti-LHCa1 can be found at the product website < https://www.agrisera.com/en/artiklar/lhca1-psi-type-i-chlorophyll-a_b-binding-protein.html>.
Validation statement for anti-Lhcb1 can be found at the product website < https://www.agrisera.com/en/artiklar/lhcb1-lhcii-type-i-chlorophyll-a_b-binding-protein-2.html>.
Validation statement for anti- Cyt b6 can be found at the product website <https://www.agrisera.com/en/artiklar/cyt-b6-petb-thylakoid-membrane-cytochrome-b6-protein-n-terminal.html>.
Validation statement for anti- CF1 β can be found at the product website < https://www.agrisera.com/en/artiklar/atpb-beta-subunits-of-atp-synthase-global-antibody.html>.
Validation statement for anti- Tic40 can be found at the product website < https://www.agrisera.com/en/artiklar/tic40-chloroplast-inner-envelope-membrane-translocon-complex-protein.html>.
Validation statement for Anti-FLAG affinity gel can be found at the product website https://www.bimake.com/product/anti-flag-affinity-gel.html>.
